# *Casp8* hypomethylation and neural tube defects in association with polycyclic aromatic hydrocarbon exposure

**DOI:** 10.1186/s13148-019-0673-6

**Published:** 2019-05-07

**Authors:** Yun Huang, Aiguo Ren, Linlin Wang, Lei Jin, Shanshan Lin, Zhiwen Li, Jasmine A. McDonald

**Affiliations:** 1 Institute of Reproductive and Child Health, National Health Commission Key Laboratory of Reproductive Health, Peking University, Beijing, 100191 China; 2 Department of Epidemiology and Biostatistics, School of Public Health, Peking University, Beijing, 100191 China; 3 Division of Birth Cohort Study, Guangzhou Women and Children’s Medical Center, Guangzhou Medical University, Guangzhou, 510623 China; 4 Department of Epidemiology, Mailman School of Public Health, Columbia University Medical Center, 722 West 168th Street, New York, NY 10032 USA; 5 Herbert Irving Comprehensive Cancer Center, Columbia University Medical Center, New York, NY 10032 USA

**Keywords:** Neural tube defects, Polycyclic aromatic hydrocarbons, Caspase-8, Hypomethylation, Apoptosis, Oxidative stress

## Abstract

**Background:**

Epidemiological studies have found that prenatal exposure to polycyclic aromatic hydrocarbons (PAHs) is associated with increased risk for neural tube defects (NTDs). Aberrant DNA methylation, excessive apoptosis, and oxidative stress have been implied as the mechanism underlying the association between PAH exposure and NTDs, respectively. However, the role of DNA methylation aberration of apoptotic initiator *CASP8* (caspase-8, apoptosis-related cysteine peptidase) in the formation of NTDs in association with PAH exposure is not known. By combining a case–control study and mouse model, we aimed to explore the full spectrum of the links from PAH exposure, oxidative stress, *CASP8* methylation change, caspase-8 activation, apoptosis, to NTD formation.

**Results:**

Hypomethylation of *CASP8* promoter was noticed in the microarray profiled by Infinium HumanMethylation450 BeadChip using neural tissues from 10 terminated NTD fetuses and 8 terminated non-malformed fetuses (14 CpG sites, with *β* difference ranging between 8.8 and 26.3%), and was validated in a larger case–control sample performed with neural tissues from 80 NTD cases and 32 non-malformed fetuses, using the Sequenom MassARRAY system (7 CpG sites). Hypomethylation of *CASP8* was a risk factor for NTDs (aOR = 1.11; 95% CI, 1.05–1.17) based on the logistic regression model. According to Pearson’s correlation, methylation levels of *CASP8* were inversely correlated with PAH concentrations in maternal serum and with oxidative stress markers in fetal neural tissues (*p* < 0.05). In the animal study, increased NTD rates (13.5% frequency), *Casp8* hypomethylation, caspase-8 upregulation, increased caspase-8 cleavage, and excessive apoptosis were found in mouse embryos cultured with benz(a)pyrene (BaP) in vitro. Antioxidant *N*-acetyl-L-cysteine (NAC) and BaP co-treatment attenuated the changes found in BaP treatment group.

**Conclusions:**

Hypomethylation of *Casp8* promoter is associated with the formation of NTDs, and *Casp8* hypomethylation may be induced by oxidative stress that resulted from exposure to PAHs.

**Electronic supplementary material:**

The online version of this article (10.1186/s13148-019-0673-6) contains supplementary material, which is available to authorized users.

## Introduction

Neural tube defects (NTDs) are a group of severe congenital malformations of the central nervous system resulting from incomplete closure of the neural tube during early embryogenesis [1]. On average, NTDs affect approximately 1 in every 1000 established pregnancies worldwide, with a higher prevalence in miscarried pregnancies [2, 3]. Annually, more than 320,000 infants are born with NTDs around the world [4]. The etiology of NTDs is complex, with contributions from environmental exposure, genetic variants, and gene–environment interactions. It is suggested that maternal exposure to polycyclic aromatic hydrocarbons (PAHs), a class of ubiquitous organic persistent pollutants, is associated with an increased risk for NTDs in humans [5–8], and an elevated occurrence of NTDs is also found in mouse embryos of dams exposed to benzo(a)pyrene (BaP), a well-studied PAH [9]. Although pathological processes, including oxidative stress [9, 10], DNA adduct formation [7], and DNA methylation dysregulation [11], are reported to be involved in PAH teratogenicity, the underlying mechanisms that cause PAH-induced NTDs remain unknown.

Spatiotemporally regulated apoptosis is a requirement for normal neural tube closure [12]. Excessive apoptosis has been found in fetal central nervous tissue of human NTD cases and murine NTDs [9, 13, 14], which indicate a role for un-regulated apoptosis in NTD formation. The activation of aspartate-specific cysteine protease (caspase)-8 acts as an initiator in the extrinsic apoptosis pathway which is initiated upon activation of death receptors [15]. Both studies in human and mice have revealed that levels of cleaved caspase-8 are significantly higher in neural tissue of NTD cases relative to controls [13, 16]. As reviewed in Das and Henkler’s article, PAH-mediated genotoxicity can trigger extrinsic apoptosis, with increases in caspase-8 [17, 18]. In previous studies, we have identified increased levels of apoptotic cells and cleaved caspase-8 in the neural tissue of NTD cases, and the percentage of apoptotic cells in fetal neural tissue was positively correlated with the concentrations of PAHs in maternal serum [9, 13]. However, it remains unknown whether excessive apoptosis is caused by exposure to PAHs via caspase-8 dysregulation.

Growing evidence has indicated that PAHs may result in DNA methylation dysregulation [19, 20], the best-known epigenetic change thought to be involved in the development of many multifactorial diseases, including NTDs [11, 21]. Exposure to PAHs leads to oxidative stress [9, 22], and oxidative stress has been reported to be associated with DNA hypomethylation, as found in human, animal, and cell studies [23–25]. A mouse study suggested that reactive oxygen species (ROS) may induce hypomethylation and subsequent upregulation of specific genes [26]. Increased levels of caspase-8 and ROS have both been detected in hyperglycemia-induced NTDs, and antioxidants could ameliorate NTD occurrence with decreased caspase-8 cleavage [14, 16]. In addition, ROS generation induced by PAHs has been identified as a mechanism of PAH-mediated apoptosis [27]. However, the full spectrum of the links from PAH exposure, oxidative stress, *CASP8/Casp8* methylation change, caspase-8 activation, apoptosis, to NTD formation has not yet been fully explored.

Therefore, we hypothesized that the decreased methylation of caspase-8 gene (*Casp8*), in association with oxidative stress induced by PAH exposure, may lead to NTDs. We tested this hypothesis by examining the methylation levels of *CASP8* in neural tissues from a small sample of NTD cases and controls, using a genomic microarray. Then, the discovered site-locus-specific methylation was validated using neural tissues from a larger sample of NTD cases and non-malformed controls. The correlations between the validated DNA methylation intensity of *CASP8* with PAH concentrations in maternal serum and with oxidative stress markers in fetal neural tissues were examined. Finally, to verify the findings in the human subject study, the CpG sites of *Casp8*, expression of caspase-8, and apoptosis levels were examined in mouse embryos exposed to BaP or BaP and antioxidant *N*-acetyl-L-cysteine (NAC) co-treatment in an in vitro whole-embryo culture model.

## Results

### *CASP8* methylation in microarray data

In the discovery stage, the methylation values of CpG sites in *CASP8* were extracted from the genome-wide methylation dataset generated using an HM450K microarray. A total of 26 CpG sites in *CASP8* were detected with the microarray, of which 22 were more hypomethylated in NTD cases compared to controls. Of these 22 sites, 14 were enriched in the promoter region and were significantly hypomethylated in NTD fetuses. For those 14 CpG sites, six CpG probes (cg09464206, cg27410837, cg04048517, cg24410214, cg19448993, and cg02878216) were located in the TSS1500 region, four (cg23882545, cg14962032, cg25748441, and cg25095814) were located in the TSS200 region, and the remaining four (cg00978584, cg20418725, cg12604794, and cg04286206) were located at 5′UTR of *CASP8* (Additional file 1: Table S4).

### Validation of *CASP8* methylation in a larger sample

In the validation stage, we examined the methylation status of CpG sites in the promoter region of *CASP8* identified in the discovery stage using Sequenom EpiTYPER in 80 NTD cases and 32 controls. The distribution of the maternal and fetal characteristics is presented in Additional file 1: Table S5. Significant differences were found in maternal education, occupation, parity, unplanned pregnancy, and periconceptional folate supplementation between the two groups. Case mothers were more likely to be less-educated farmers, to have higher parity, and to have had periconceptional folate supplementation than control mothers. A significantly lower proportion of case women (38.0%) reported unplanned pregnancies, compared to 74.2% of control women. No significant differences were found between the case and control groups for any other characteristics.

**Fig. 1:**
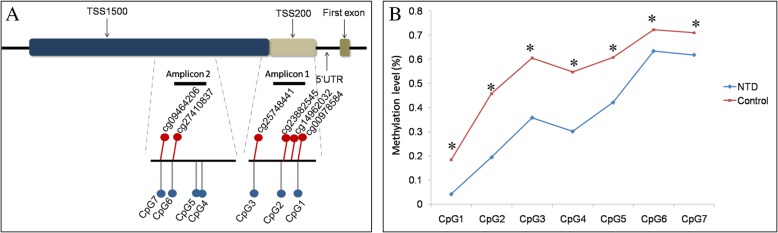
Location and methylation levels of CpG sites in *CASP8* examined in the discovery and validation stage in human subjects. **a** Locations of the CpG sites detected by HM450K and Sequenom EpiTYPER. The differentially methylated CpG units identified by HM450K Array are indicated as red dots above the black line. CpG sites detected in the validation stage are indicated as blue dots under the black line. **b** Methylation patterns of the *CASP8* gene detected with Sequenom EpiTYPER in a larger sample of human subjects. Methylation levels for each CpG unit between NTD cases and controls within the amplicons were analyzed. HM450K, HumanMethylation450 BeadChip; NTD, neural tube defect. *Methylation levels of CpG sites were significantly different between the case and control groups (all *p* < 0.05)

We designed two DNA amplicons to validate the CpG sites discovered in stage one (Fig. 1a). Significantly hypomethylated CpG sites with a *β* difference (absolute value) greater than 0.2 were chosen as a priority for DNA amplicon design. Amplicon 1 covered three CpG sites (CpG1 to CpG3), in which CpG3 was the same as the hypomethylated CpG site of cg25748441 identified in the discovery stage. Amplicon 2 covered four CpG sites within the TSS1500 region of CASP8 (CpG4 to CpG7), in which CpG6 and CpG7 were the same as the cg27410837 and cg09464206 CpG sites, respectively, which were identified as differentially hypomethylated in the discovery stage.

All of the seven detected CpG sites were significantly hypomethylated in NTD cases, including three HM450K array-matched CpG sites (Fig. 1b). After Bonferroni correction, all of the sites demonstrated significant hypomethylation in NTD cases with a *β* difference ranging between 8.8% and 26.3%, reaffirming hypomethylated status in the promoter region of *CASP8* in NTD cases. The details are shown in Additional file 1: Table S6.

The NTD group showed a significantly lower average methylation level of *CASP8* (36.8%) than the control group (54.7%). Hypomethylation of *CASP8* was a risk factor for NTDs (aOR = 1.11; 95% CI, 1.05–1.17) according to the logistic regression model, which indicated that the risk for NTDs increased by 10.8% with every 1% reduction of the average methylation level of *CASP8*. Risk for two major subtypes of NTDs, namely, anencephaly and spina bifida, was also associated with hypomethylated *CASP8* (Table 1).

**Table 1:** Associations between mean methylation level of the *CASP8* gene and risk of NTDs

Group	*n*	Mean methylation (% ± SE)	*p* value	OR (95% CI)	Adjusted-OR (95% CI)^*a*^
Total NTDs ^*b*^	80	0.368 (0.012)^*c*^	< 0.001	1.10 (1.06, 1.14)	1.11 (1.05, 1.17)
Anencephaly	33	0.381 (0.018)	< 0.001	1.08 (1.04, 1.13)	1.11 (1.03, 1.19)
Spina bifida	39	0.359 (0.018)	< 0.001	1.09 (1.04, 1.13)	1.11 (1.04, 1.18)
Controls	32	0.547 (0.031)	–	1.0	1.0

Note: *SE* standard error, *OR* odds ratio, *CI* Confidence interval, *NTDs* neural tube defects

^*a*^Maternal general characteristics and exposure, including educational level, occupation, parity, unplanned pregnancy, and periconceptional folate supplementation, which were unevenly distributed between the case and control groups, were adjusted in the logistic regression model

^*b*^Subtype analyses were not performed in eight encephalocele cases

^*c*^To more easily explain the regression coefficient, we took the negative centuplicate value of the methylation data into the model

### PAH exposure and *CASP8* methylation

In a previous study, we found that levels of PAHs were significantly higher in maternal serum of NTD cases than in controls [6]. In the present study, we determined the correlation between the methylation status of *CASP8* in fetal neural tissue and PAH levels in maternal serum. The methylation level of *CASP8*_CpG_2 showed an inverse correlation with total PAHs. Three CpG sites and the average methylation level of *CASP8* were inversely correlated with the concentration of H_PAHs. The remaining four CpG sites also tended to show an inverse correlation with H_PAHs, although statistical significance was not reached (Table 2). A subgroup analysis was made in NTD cases Additional file 1: Table S7; the results also indicated an inverse correlation between concentration of H_PAHs and *CASP8* methylation in the NTD group.

**Table 2:** Correlation analysis of differentially methylated CpG sites and PAH concentrations in maternal serum

CpG sites	*N*	Total PAHs, ng/g lipid		L_PAHs, ng/g lipid		H_PAHs, ng/g lipid	
		*ρ*	*p*	*ρ*	*p*	*ρ*	*p*
*CASP8*_CpG_1	56	− 0.242	0.072	− 0.221	0.102	− 0.275	0.040
*CASP8*_CpG_2	58	− 0.275	0.037	− 0.257	0.051	− 0.290	0.027
*CASP8*_CpG_3	58	− 0.212	0.109	− 0.182	0.171	− 0.255	0.053
*CASP8*_CpG_4	58	− 0.250	0.058	− 0.212	0.111	− 0.296	0.024
*CASP8*_CpG_5	58	− 0.165	0.216	− 0.125	0.352	− 0.228	0.086
*CASP8*_CpG_6	58	0.011	0.937	0.061	0.650	− 0.101	0.450
*CASP8*_CpG_7	56	− 0.157	0.247	− 0.136	0.319	− 0.201	0.137
*CASP8*_average	58	− 0.239	0.071	− 0.199	0.134	− 0.299	0.023

Note: PAHs, polycyclic aromatic hydrocarbons; total PAHs, sum of all PAHs; L_PAHs, sum of low-molecular-weight PAHs, including acenaphthylene, acenaphthene, fluorene, phenanthrene, anthracene, fluoranthene, and retene; H_PAHs, sum of high-molecular-weight PAHs, including pyrene, benz[a]anthracene, chrysene, benzo[b]fluoranthene, benzo[k]fluoranthene, and benzo[a]pyrene; *ρ*, Pearson’s correlation coefficient.

### Oxidative stress and *CASP8* methylation in human

Correlation analysis of oxidative stress markers and the methylation status of CpG sites in *CASP8* promoter in fetal neural tissues was performed. The methylation levels of several CpG sites were inversely correlated with antioxidant indicators GPx (CpG6, CpG7) and SOD (CpG7). One CpG site was inversely correlated with PC, a marker of protein oxidation (Table 3). A subgroup analysis was made in NTD cases (Additional file 1: Table S8), and the results indicated an inverse correlation between GPx (CpG6, CpG7, and *CASP8*_average) and *CASP8* methylation in the NTD group.

**Table 3:** Correlation analysis of oxidative stress markers in fetal neural tissues and differentially methylated CpG sites in *CASP8*

CpG sites	*N*	SOD, unit/mg protein		GPx, unit/mg protein		TAC, unit/mg protein		MDA, nmol/mg protein		PC, nmol/mg protein	
		*ρ*	*p*	*ρ*	*p*	*ρ*	*p*	*ρ*	*p*	*ρ*	*p*
*CASP8*_CpG_1	31	− 0.078	0.675	− 0.225	0.224	0.023	0.903	0.103	0.582	− 0.423	0.018
*CASP8*_CpG_2	31	− 0.005	0.980	− 0.091	0.627	0.038	0.840	0.093	0.618	− 0.266	0.148
*CASP8*_CpG_3	31	− 0.023	0.903	− 0.202	0.277	0.128	0.491	0.100	0.591	− 0.313	0.086
*CASP8*_CpG_4	31	− 0.128	0.493	− 0.186	0.317	0.022	0.907	0.073	0.696	− 0.256	0.164
*CASP8*_CpG_5	31	− 0.068	0.716	− 0.233	0.207	0.037	0.842	0.136	0.467	− 0.185	0.320
*CASP8*_CpG_6	31	0.006	0.976	− 0.402	0.025	0.144	0.440	0.183	0.323	− 0.029	0.875
*CASP8*_CpG_7	30	− 0.386	0.035	− 0.542	0.002	0.284	0.128	0.352	0.057	− 0.228	0.226
*CASP8*_average	31	− 0.114	0.540	− 0.265	0.149	0.110	0.555	0.132	0.478	− 0.266	0.148

Note: *SOD* superoxide dismutase, *GPx* glutathione peroxidase, *TAC* total antioxidant capacity, *MDA* malondialdehyde, *PC* protein carbonyl, *ρ* Pearson’s correlation coefficient

### *Casp8* methylation and expression in BaP-treated mouse embryos

To validate the level of *Casp8* methylation in association with BaP exposure, a whole-embryo culture model was used. E8.5 embryos were explanted from the uterus of pregnant mice and cultured in rat serum with DMSO or BaP treatment. After 48 h of in vitro culture, the embryos developed into a stage where the neural tube would have closed under normal conditions. The embryos were collected and inspected under a dissecting microscope. The diameter of the yolk sac, the length of the head, and the crown–rump length of the mouse embryos cultured in BaP medium were smaller (*p* < 0.05) than those of the DMSO vehicle group (Additional file 2: Figure S1). Embryos treated with BaP showed a significantly higher rate of NTDs (13.5%) than the vehicle group (*p* < 0.05; Additional file 2: Figure S2 and Additional file 1: Table S9).

**Fig. 2:**
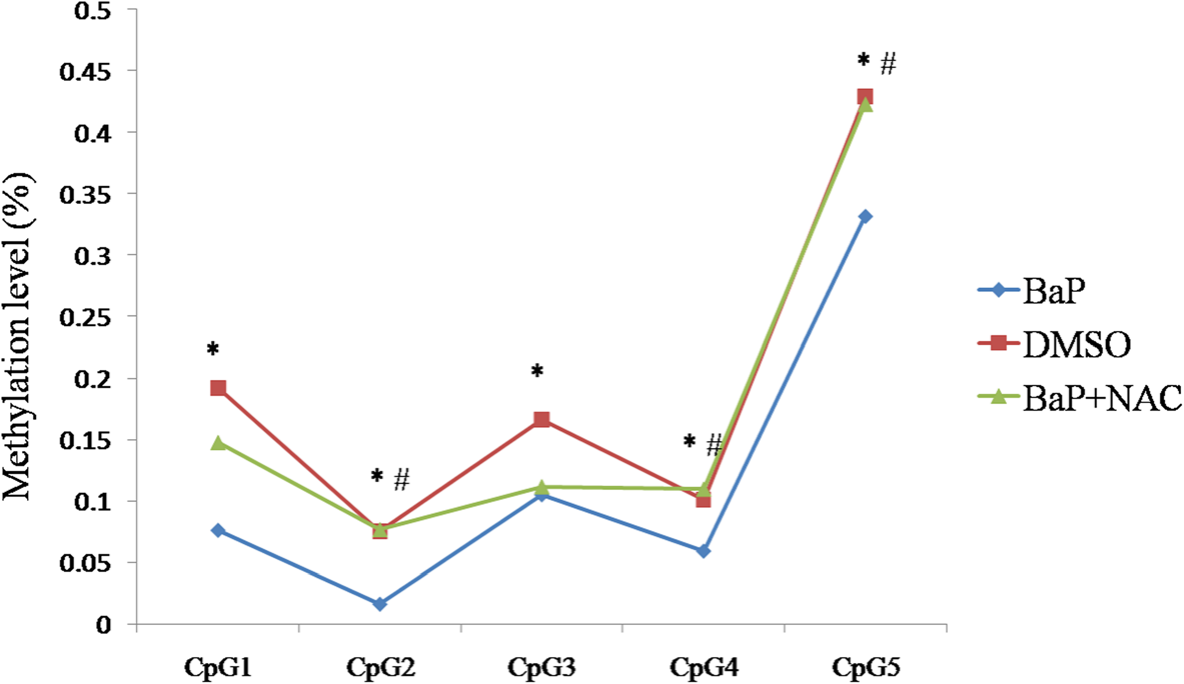
Methylation levels of CpG sites in *Casp8* examined in neural tissue of mouse embryos. The methylation levels for each CpG unit in the DMSO, BaP, and BaP + NAC groups within the amplicons were analyzed. DMSO, dimethylsulphoxide; BaP, benz(a)pyrene; NAC, *N*-acetyl-L-cysteine. ^*^Significant difference between DMSO and BaP group (*p* < 0.05). ^#^Significant difference between BaP and BaP + NAC (*p* < 0.05)

To examine whether *CASP8* hypomethylation, which was detected in the promoter region of human NTD cases, was also present in BaP-treated mouse embryos, we amplified one DNA amplicon in the promoter of *Casp8* in mouse neural tissue. All of the five detected CpG sites were significantly hypomethylated in BaP-treated mouse embryos (Fig. 2). After Bonferroni correction, two CpG sites still demonstrated significant hypomethylation in the BaP group, with *β* differences of 11.6% and 4.2%, respectively, suggesting that decreased methylation of *Casp8* was present in BaP-treated mouse embryos which had increased incidence of NTDs. The details are represented in Additional file 1: Table S10.

**Fig. 3:**
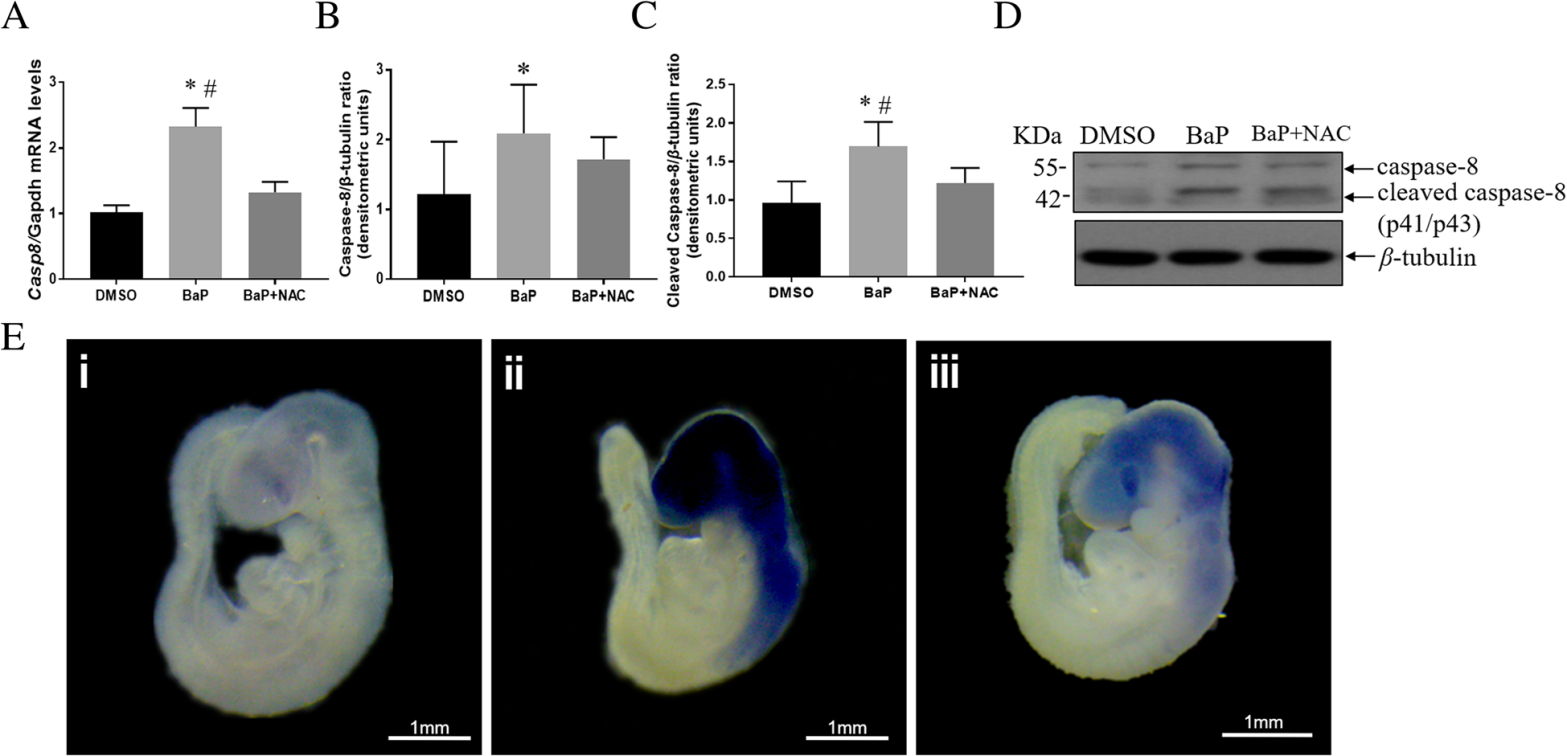
Effects of BaP and NAC on mRNA and protein expressions of caspase-8. **a** Relative expression of *Casp8* mRNA of E10.5 embryos exposed to BaP and co-exposed to NAC by real-time PCR. Data for *Casp8* mRNA were normalized by *Gapdh* for each sample (mean ± SE; *n* = 5). **b** Expression of caspase-8 protein in E10.5 embryos exposed to BaP and co-exposed to NAC by western blot. Data for caspase-8 protein were normalized against *β*-tubulin and are represented as mean ± SD to that of control (*n* = 7). **c** Expression of cleaved caspase-8 protein in E10.5 embryos exposed to BaP and co-exposed to NAC by western blot. Data for cleaved caspase-8 protein were normalized against *β*-tubulin and are represented as mean ± SD to that of control (*n* = 7). **d** Representative western blot images of *Casp8* and *β*-tubulin expressions. **e** Representative images of expression patterns of *Casp8* in E9.5 mouse embryos measured using whole-mount *in situ* hybridization. (i) Control, (ii) BaP exposure, and (iii) BaP + NAC. Bar = 1 mm. Error bars represent standard error or deviation of the mean. BaP, benz(a)pyrene; NAC, *N*-acetyl-L-cysteine; SE, standard error; SD, standard deviation. ^*^p < 0.05 vs. control; ^#^*p* < 0.05 vs. NAC co-treatment

Since hypomethylation is likely to induce gene upregulation, we analyzed the mRNA levels and protein abundance of caspase-8 in neural tissue of BaP-treated mouse embryos. Consistent with decreased methylation levels in *Casp8* promoter, the mRNA expression of *Casp8* in the BaP-treated group was significantly higher than that in DMSO controls, according to real-time PCR (*p* < 0.05; Fig. 3a). Whole-mount in situ hybridization of *Casp8* showed specific staining of neural tissues, particularly in the forebrain, midbrain, and hindbrain of BaP-treated E9.5 embryos (Fig. 3e). Western blot showed that the protein abundance of caspase-8 was much higher in BaP-treated embryos than in control embryos (Fig. 3b and d). These results revealed upregulation of caspase-8 in BaP-treated mouse embryos coupled with *Casp8* promoter hypomethylation.

### Effect of antioxidant on *Casp8* methylation and expression

To further examine the role that oxidative stress may have played in *Casp8* methylation during NTD formation, NAC, a known antioxidant, was added to BaP medium at the beginning of in vitro embryo culture. The diameter of the yolk sac, head length, and crown–rump length all grew better in mouse embryos in the NAC and BaP co-treated group than those in the BaP group, with head length being restored to the same level as the vehicle group (Additional file 2: Figure S1). NAC rescued BaP-induced NTDs in vitro (*p* < 0.05; Additional file 1: Table S7).

We detected the methylation status of *Casp8* in mouse embryos co-treated by NAC and BaP to examine whether antioxidant could rescue the hypomethylation caused by BaP. Three of the five CpG sites detected showed higher methylation levels in the group co-treated with NAC than in the BaP group (Fig. 2), one of which still showed significance after Bonferroni correction. These results suggested that *Casp8* hypomethylation caused by BaP could be rescued by antioxidation. More details are represented in Additional file 1: Table S8.

Caspase-8 expression was also analyzed in the neural tissue of mouse embryos co-treated with NAC and BaP. The elevated mRNA expression of *Casp8* induced by BaP was normalized by NAC co-treatment (*p* < 0.05; Fig. 3a and d). Consistent with the results of real-time PCR, NAC mitigated the expression of *Casp8* in the brain and spinal cord according to whole-mount in situ hybridization assays. Co-exposure to NAC also attenuated the increased procaspase-8 protein expression induced by BaP exposure (Fig. 3b and d). Together, these findings revealed that antioxidation could rescue the upregulation of caspase-8 accompanied by *Casp8* hypomethylation induced by BaP exposure.

### Effects of BaP and antioxidant on apoptosis

**Fig. 4:**
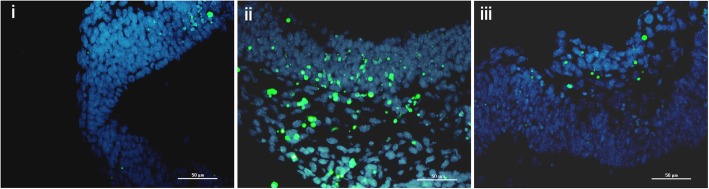
Effects of BaP and NAC on apoptosis level of E10.5 embryos. Representative images of TUNEL-positive cells (green) and 4′,6-diamidine-2-phenylidoledihydrochloridestain cells (blue). Slices were sectioned along a coronal plane of mouse embryos. **i** Control, **ii** BaP exposure, and **iii** BaP + NAC. Bar = 50 μm. BaP, benz(a)pyrene; NAC, *N*-acetyl-L-cysteine

Since caspase-8 is thought to be the initiator caspase, we further hypothesized that overexpression of *Casp8* resulting from BaP exposure would lead to excessive apoptosis. Hence, we measured the protein level of cleaved caspase-8 by western blot and detected apoptosis levels in embryos using TUNEL assays. Levels of cleaved caspase 8 were significantly upregulated after BaP exposure, while NAC treatment significantly suppressed BaP-induced caspase 8 cleavage (Fig. 3c and d). Apoptotic cells were more frequently observed in BaP-treated embryos than in controls (*p* < 0.05), while only a few TUNEL-positive cells were observed in mice co-treated with NAC and BaP as in the DMSO group (Fig. 4 and Additional file 2: Figure S3 and S4). These results suggest that antioxidation could alleviate apoptosis induced by BaP in mouse embryos.

## Discussion

Increased caspase-8 expression and concomitant excessive apoptosis have been reported in PAH-related NTDs [9, 13], while the underlying mechanisms are not well studied. Under this context, we focused on the aberration of DNA methylation, one of the mechanisms behind gene–environment interaction. In this study, *CASP8* hypomethylation was discovered in microarray data obtained from a small number of human NTD cases and controls and subsequently validated in a larger independent case–control cohort. The methylation levels of *CASP8* were inversely correlated with concentrations of PAHs in maternal serum and with oxidative stress markers in fetal neural tissues. In further validation, increased NTD rates, *Casp8* hypomethylation, upregulation of caspase-8, increased cleaved caspase-8, and excessive apoptosis were found in BaP-treated mouse embryos cultured in an in vitro whole-embryo culture model. Antioxidant NAC attenuated the changes induced by BaP in mouse embryos.

Since NTD formation is thought to be associated with elevated activation of caspase-8, which plays a crucial role in the initiation of extrinsic apoptosis [13, 15], we detected the methylation level of *CASP8* promoter in neural tissue from human NTD cases and controls. After exploring the HM450K microarray data and validating the CpG sites with a larger case–control cohort, we found that both average and locus-specific methylation levels in the promoter region of *CASP8* were significantly decreased in human NTDs. According to a logistic regression model, the hypomethylation of *CASP8* was a risk factor for NTDs (aOR = 1.11; 95% CI, 1.05–1.17), after adjusting for potential confounders. Generally, although not exclusively, hypomethylation of the gene promoter region is associated with gene upregulation [28]. Supportively, in our study, upregulated expression of mRNA and protein of caspase-8 and increased cleaved caspase-8 were found in mouse embryos that showed an increased incidence of NTDs accompanied with *Casp8* promoter hypomethylation in neural tissue after BaP exposure. The increased caspase-8 and cleaved caspase-8 found in our animal study are supported by previous studies that have found that elevated caspase-8/cleaved caspase-8 levels in human fetuses are associated with increased risk for NTDs [13, 16]. We further found elevated apoptosis levels in mouse embryos, which showed a decreased methylation level of *Casp8* promoter after BaP exposure. The deregulation of apoptosis has been implicated in the development of NTDs [2]. Although apoptosis is needed in the closure of the neural tube for the dorsolateral hinge points’ formation and neural folds remodeling, excessive apoptosis may hinder the participation of cells in fusing neural folds or may disrupt the physical continuity of the dorsal midline, leading to NTDs [29]. To the best of our knowledge, no earlier studies have explored the role that aberrant DNA methylation of apoptosis-related genes may play in NTD formation, neither in human subjects nor in animals. These results suggest that *Casp8* hypomethylation may induce excessive apoptosis by upregulating caspase-8 expression, leading to NTD formation. Our findings provide insight into the role of aberrant DNA methylation in abnormal neurulation during the development of human NTDs.

In previous studies, we found higher levels of cleaved caspase-8 in NTD cases than in controls, and case mothers have higher levels of PAH exposure than control mothers [13]. Therefore, we analyzed the relationship between PAH levels in maternal serum and locus-specific methylation levels of *CASP8* in neural tissue of NTD fetuses. The concentration of H_PAHs tended to be inversely correlated with all of the CpG sites and with the average methylation level of *CASP8*. To explore the association between PAH exposure and aberrant DNA methylation further, we treated mouse with BaP during neural tube closure. Significant lower locus-specific methylation levels were detected in the promoter of *Casp8* in neural tissue of mouse embryos in the BaP-treated group, which also showed an increased NTD rate.

Evidence regarding the effect of PAH exposure on *Casp8* methylation has not yet been retrieved. For global DNA methylation, the association between PAH exposure and decreased DNA methylation has been reported both in human and animal studies [19, 30, 31]. PAH metabolites in maternal urine are associated with decreased global methylation status in a Chinese cohort [32]. Several in vitro studies have found that BaP exposure could decrease global DNA methylation in a dose-response manner [33, 34]. However, the associations between PAH exposure and DNA methylation in specific genes were inconsistent in human, animal studies, and in vitro studies [11, 31, 35, 36]. For example, a case–control study conducted in China reported positive correlations between PAH levels in maternal serum and methylation levels of tight junction pathway genes CTNNA1 and MYH2 in neural tissue from NTD cases [11], while LINE-1 and MGMT genes were found to be hypomethylated in 82 PAH-exposed coke-oven workers compared to 62 unexposed controls, and gene-specific methylation of LINE-1 and MGMT was inversely correlated with levels of urinary PAH metabolites [31]. These studies indicate that PAHs may contribute to the methylation of distinct genes differently.

The mechanism by which PAH exposure causes aberrant DNA methylation during NTD formation remains unclear. Since oxidative stress has been implicated in the etiology of NTDs [9, 10, 37] and has been reported to induce DNA hypomethylation in congenital anomalies [23, 38], we analyzed the associations between markers of oxidative stress and the methylation levels of locus-specific CpG sites in fetal neural tissues. Inverse correlations were found between antioxidant indicators or protein oxidation markers and the methylation status of *CASP8* in NTD cases. To further explore the role of oxidative stress in BaP-induced DNA hypomethylation in NTD formation, we cultured mouse embryos in BaP with and without NAC. The incidence of NTDs was significantly lower in the NAC co-treated group than in the BaP-treated group. NAC also partially restored the decreased methylation level of *Casp8* to that of the control group. NAC is a kind of thiol and acts as a precursor in the synthesis of glutathione, which plays a vital role in cellular defense against oxidative stress. NAC has also been reported to ameliorate global DNA hypomethylation in arsenic-induced NTDs in chick embryos [38]. A randomized trial conducted in Vienna investigated the impact of an antioxidant- and vitamin-rich diet on the epigenetic pattern of *MLH1* in patients with non-insulin-dependent diabetes mellitus type 2 and impaired fasting glucose. In support of our study, this study found that antioxidant intervention increased the methylation level in promoter of *MLH1*, and the DNA strand breaks induced by oxidative stress showed an inverse correlation with methylation level of *MLH1* [39].

Oxidative stress has been suggested as one of the possible mechanisms through which PAHs exert its effect on DNA methylation regulation [40]. A representative example of oxidative DNA damage, 8-hydroxy-2′-deoxyguanosine (8-OHdG), which is also increased in maternal serum in NTD cases [10], may suppress human DNA methyltransferase (DNMT) and murine *Dnmt3a* and inhibit DNA methylation by downregulating the methylation of nearby cytosine base [40, 41]. Moreover, oxidative stress may lead to the depletion of *S*-adenosyl-L-methionine (SAM), a methyl donor for methylation reaction under the catalysis of DNMT, which would reduce DNA methylation [42]. Further, increased ROS may also upregulate ten-eleven translocation (TET), an enzyme that induces demethylation, resulting in DNA hypomethylation [43]. The inverse correlation between oxidative markers and *CASP8* methylation levels in human and the successful rescue of *Casp8* hypomethylation with NAC in mouse agrees with our hypothesis that PAHs may affect the methylation of *CASP8/Casp8* by inducing oxidative stress during NTD formation.

To the best of our knowledge, few studies have detected the methylation status of DNA directly in neural tissue of NTD cases. In consideration of the tissue specificity of DNA methylation, using central neural tissue from specific lesion sites is optimal in epigenetic studies of NTD etiology. However, the limitations of the present study should be mentioned. DNA methylation is time specific. The human tissues we collected were mostly at second trimester, while neural tube closure is completed at the end of the fourth-week post-fertilization, corresponding to about 6 weeks of clinical gestation. However, in our animal study, the neural tissue that we used to examine the methylation status was from mouse embryos that had just completed neural tube closure. In addition, we had no transcript-expression data for the *CASP8* gene in human subjects since it is difficult to obtain tissues that are fresh enough from terminated NTD cases to extract mRNA. However, information on the expression of caspase-8 from the in vitro mouse model allows insight from a different point of view. In addition, although the promoter region is the most studied region that is known to play a decisive role in the regulation of gene activity, DNA methylation function varies in different genomic contexts—for example, methylation in gene body may stimulate, but in TSS may block, transcription [44]. In this study, we only examined CpGs located in the promoter region and did not explore methylation changes in gene body and other regulatory regions. A different gene region should be taken into consideration in future studies.

## Conclusion

Hypomethylation of the promoter in *CASP8* is associated with an increased risk of NTDs in human subjects, and this association was confirmed in mice that were exposed to BaP. The decrease in the methylation status of *CASP8*/*Casp8* may be related to oxidative stress induced by PAH exposure. These findings provide novel insights into the role of epigenetic modifications of the *Casp8* in NTD formation in association with exposure to PAHs.

## Methods

### Human subjects

The enrollment of human subjects has been described in a previous report [5]. Briefly, human subjects were recruited from an ongoing population-based birth-defects surveillance system that was conducted in five rural counties in Shanxi Province of northern China between 2011 and 2014, where NTD prevalence is among the highest in the world, 31.5 per 10,000 births in 2014 [45]. For cases, fetuses electively terminated following prenatal diagnosis of an NTD were included. For controls, terminated fetuses with no congenital malformations were included. Information on sociodemographic characteristics, reproductive history, lifestyle, folic acid supplementation, active smoking and exposure to passive secondhand smoke, and fuel used for cooking and heating during the periconceptional period were collected through in-person interviews by trained local healthcare workers. Samples of maternal venous blood were collected at delivery or pregnancy termination, temporarily stored at − 20 °C at local hospitals, and then transferred to our laboratory on dry ice and stored at − 80 °C until use. Spinal cord and brain tissue samples were collected from terminated NTD and non-malformed fetuses by experienced pathologists and were immediately stored at − 80 °C until the analyses were performed. The study protocol was approved by the Institutional Review Board of Peking University, and written informed consent was obtained from all participating women.

### Animals and whole-embryo culture

Primigravida CD-1 (ICR) mice (ages 8–12 weeks) were used in the experiment. Female mice were mated with males at 8 AM, and vaginal plugs were examined 4 h later. 10 AM on the day of finding a vaginal plug was considered 0 day of embryonic development (E0). Animals were cared for by the laboratory animal center at Peking University Health Science Center under a 12-h light/dark cycle in a temperature/humidity controlled facility and were allowed free access to food and water.

Whole-embryo culture was performed following the procedures described in a previous study [46]. Briefly, pregnant mice were sacrificed via cervical dislocation on E8.5. Only embryos with three to eight somites were utilized. Decidua and Reichert’s membranes were removed before the embryos were randomly placed into sealed culture bottles (3–5 embryos per bottle) that contained rat serum with 1‰ dimethylsulphoxide (DMSO), 5 mM BaP (Sigma-Aldrich), or 5 mM *N*-acetyl-l-cysteine (NAC) and 5 mM BaP, as determined by preliminary experiments. Then, the embryos were incubated at 37 °C in sealed culture bottles fitted into a device that were rotated at 25 rpm. The culture bottles were initially gassed with a mixture of 5% O_2_, 5% CO_2_, and 90% N_2_ for 2.5 min. Subsequent gassing was performed after 16 h (20% O_2_, 5% CO_2_, and 75% N_2_) and 10 h (40% O_2_, 5% CO_2_, and 55% N_2_). Embryos used for ROS detection and whole-mount in situ hybridization were cultured for 24 h before being collected; the rest were cultured for 48 h. Neural tissue was isolated from the embryos under a dissecting microscope and immediately put into liquid nitrogen before being stored at − 80 °C for further use. The study protocol was approved by the Institutional Animal Care and Use Committee of Peking University (certificate no. LA2013–36).

### Methylation assay

Methylation assays on DNA from human subjects were performed in two stages. In the first (i.e., the discovery stage), we used Infinium HumanMethylation450 BeadChip (HM450K; Illumina, San Diego, CA, USA) to profile genome-wide DNA methylation, using neural tissue from 10 NTD cases and 8 non-malformed control fetuses. In the second stage (i.e., the validation stage), neural tissues from 80 NTD cases and 32 non-malformed control fetuses were used to validate the results in the discovery stage by using the Sequenom MassARRAY system, as previously described elsewhere [11].

DNA was extracted from fetal neural tissue using the QIAamp DNA MiniKit (QIAGEN, Hilden, Germany). The concentration of DNA was measured with a NanoDrop2000 Ultramicro spectrophotometer (Thermo Fisher Scientific, MA, USA). All of the DNA samples were stored at − 80 °C until used for assays. Bisulfite conversion of 500 ng DNA was conducted with an EZ DNA Methylation Kit (Zymo Research, CA, USA), following the manufacturer’s instructions. To minimize the potential bias caused by variable conversion efficiency, the bisulfate conversion reaction was performed in duplicate for each sample, and the bisulfate-treated DNA was pooled for subsequent array analyses. Genome-wide DNA methylation was performed using the HM450K according to the manufacturer’s protocol. Methylation array data for *CASP8* were extracted from the genome-wide microarray.

In the validation stage, target verification of differentially methylated CpGs in the promoter of *CASP8* was performed using the Sequenom EpiTYPER (Sequenom, San Diego, USA). Bisulfate-treated DNA was amplified via PCR. The PCR primers of *CASP8* were designed using the online tool EpiDesigner (http://www.epidesigner.com/). We designed two amplicons to cover the six aberrant CpG sites, for which the differences between cases and controls were larger than 0.2 in the HM450K array. The primer sequence is presented in Additional file 1: Table S1). About 20 ng of bisulfate-treated DNA was amplified by PCR. Enzyme digestion was performed using T cleavage enzyme (T Cleavage Mix) after the PCR product was incubated with shrimp alkaline phosphatase. Then, the product was desalinized using 384 dimple plates before being detected by the MassARRAY system. EpiTYPER Analyzer software version 1.0 was used to analyze the methylation levels of DNA. Positive controls (complete methylation) and negative controls (non-methylation) were used for quality control in methylation assay.

Mouse DNA methylation assay was also performed in Sequenom EpiTYPER (Sequenom, San Diego, USA). DNA was extracted from neural tissue of mouse embryos cultured with DMSO, BaP, or NAC and BaP co-treatment using the QIAamp DNA MiniKit (QIAGEN, Hilden, Germany). A total of 500 ng genomic DNA from each sample was treated with bisulfite using the EZ DNA Methylation Kit (Zymo Research, CA, USA). Because *Casp8* may have multiple transcripts, to validate the differentially methylated CpGs found in the promoter of human *CASP8* in mouse tissue, we designed amplicons within the promoter of mouse *Casp8* that regulate the exons that encode proteins sharing a high homology with the transcript discovered in the human methylation assay. PCR primer of *Casp8* was designed using the online tool EpiDesigner (http://www.epidesigner.com/). The primer sequence is presented in Additional file 1: Table S2.

### PAHs analysis

The detailed methods for PAHs analysis have been described elsewhere [6]. Briefly, concentrations of PAHs in maternal serum were determined using an Agilent 7890A-5975C gas chromatograph and mass spectrometer equipped with an HP-5MS capillary column (30 m × 0.25 mm × 0.25 μm). Two procedure blanks and a reagent blank were included for each batch. Case–control status of the samples was masked during chemical analysis. PAH concentration is expressed on a lipid weight basis and is reported as nanogram per gram lipid. Low-molecular-weight PAHs (L_PAHs) with two or three benzene rings and high-molecular-weight PAHs (H_PAHs) with four or five benzene rings were used as sum respectively to indicate maternal PAH exposure.

### Oxidative stress evaluation

Antioxidant indicators and oxidative damage markers in fetal neural tissues were determined according to the kit specifications (Nanjing Jiancheng Bioengineering Institute, Nanjing, China). Activity levels of superoxide dismutase (SOD), glutathione peroxidase (GPx), and total antioxidant capacity (TAC) were used as antioxidant indicators, and the contents of malondialdehyde (MDA) and protein carbonyl (PC) were used as indicators of lipid and protein oxidation, respectively.

### Real-time PCR

RNA was isolated from the neural tissue of E10.5 embryos using Trizol (Invitrogen). DNase I digestion (DNA-free, Ambion) was used to remove genomic DNA, after which RNA was reverse-transcribed using random hexamers (Superscript VILO cDNA synthesis kit). The abundance of *Casp8* mRNA was analyzed using real-time PCR (iTaqTM Universal SYBR Green Supermix, BioRad) on a 7500 Fast Real Time PCR system (Applied Biosystems), with each sample analyzed in triplicate. The primer sequence is presented in Additional file 1: Table S3. The relative quantification of each level of gene expression was normalized according to *Gapdh* expression.

### Whole-mount in situ hybridization

Whole-mount in situ hybridization of E9.5 mouse embryos was carried out using a protocol derived from previously published methods [47]. Briefly, *Casp8* probe was cloned by RT–PCR into pGEM-T (Promega) and used to generate digoxygenin-labeled cRNA probes by reverse transcription with T7 RNA polymerase (Roche). For detection, embryos were fixed in 4% paraformaldehyde at 4 °C overnight before being dehydrated through a graded series of methanol/PBT (25%, 50%, 75%, and 100% methanol in PBST) and stored at − 20 °C for future detection. After rehydration, the embryos were incubated in bleached 4:1 PBST:30% hydrogen peroxide for 1 h on ice before being digested in 10 μg/mL proteinase K. Then the preheated hybridization buffer was used to prehybridize the embryos for 1 h at 70 °C. After that, embryos were hybridized overnight in 1 μg/mL DIG-labeled RNA probe for *Casp8* and washed with Solution I (50% formamide, 5× SSC pH 5, 1% SDS) and Solution II (0.5 M NaCl, 10 mM Tris HCl pH 7.5, 0.1% Tween 20) the next day at 70 °C. Next, the embryos were pre-blocked in 10% sheep serum before being incubated in anti-Digoxigenin-AP antibody (1:2000, Roche) in 1% sheep serum overnight at 4 °C. Color detection was carried out using a developing solution (NBT/BCIP, Roche) in the dark, and the signal was captured periodically via a dissecting microscope.

### Western blot

Protein was extracted on ice from embryos at E10.5 using RIPA buffer. Protein concentrations were determined using the Bradford assay. Western blot was performed using conventional methods, with 50-μg protein run per sample on NuPAGE 4–12% Bis-Tris gel (Life technologies), followed by transfer to PVDF membranes (XCell II Blot Module, Invitrogen). The primary antibodies were rabbit anti-caspase-8 (1:500, Proteintech, Chicago, USA) and anti-*β*-TUBULIN (1:10,000, Proteintech, Chicago, USA). After incubation with secondary antibody (1:10,000, DAKO), blots were developed using ECL Prime (GE Healthcare Life Sciences) or ECL Western Blotting Substrate (Promega). ImageJ was used to detect densitometry. The results were normalized using *β*-TUBULIN loading control. Independent experiments were carried out three to four times per sample.

### TUNEL assay

Paraffin slices of E10.5 mouse embryos were used for apoptotic cell injury assays using the one-step TUNEL kit according to the manufacturer’s instruction (Beyotime Institute of Biotechnology). Briefly, the slices were dewaxed with xylene before being incubated with proteinase K, followed by TUNEL reaction agents for 1 h at 37 °C. Under a microscope (at × 400 magnification), cells with green fluorescence were defined as apoptotic cells. Five fields were randomly selected from each section, and all cells were successively counted for each field. The ratio of the number of TUNEL-positive cells to the total number of cells was determined.

### Statistical analysis

During the discovery stage, Illumina Genome Studio software (Illumina, CA, USA) was used to extract signal intensities for each probe, perform initial quality-control checks, and estimate *β*-scores and detection *p* values. The *β*-score was defined as the proportion of total signal from the methylation-specific probe or color channel. The detection *p* value was calculated as 1 – *p* value computed from the background model characterizing the chance that the target sequence signal could be distinguished from the negative controls. Independent *t* tests were used to identify differentially methylated CpG sites of *CASP8* between cases and controls. Correction for multiple comparisons was performed using the Bonferroni procedure. Differentially methylated CpG sites were identified by the following criteria: false discovery rate *p* < 0.05 and absolute *β*-value difference > 0.2.

Within the validation stage, the *χ*^2^ test or Fisher’s exact test was used to compare demographic information between the case and control groups. The difference in methylation intensity of *CASP8* between cases and controls was tested using the Shapiro–Wilk test. The independent *t* test was used to identify differentially methylated CpG sites in *CASP8*. The association between the methylation levels of CpG sites in *CASP8* and risk of NTDs was estimated from the odds ratio (OR) with 95% confidence interval (CI), using an unconditional logistic model. To better explain the coefficient, we used the negative centuplicate *β*-score in the model. We adjusted for general maternal characteristics and exposure, including educational level, occupation, parity, unplanned pregnancy, and periconceptional folate supplementation, which were unevenly distributed between the case and control groups. Correlation analyses of differentially methylated CpG sites and PAH levels in maternal serum as well as oxidative stress markers in fetal neural tissues were performed by Pearson’s correlation coefficient.

In the mouse study, the rate of NTDs was analyzed using Pearson’s *χ*^2^ test. Differentially methylated CpG sites of *Casp8* in neural tissues among fetal mice with DMSO, BaP, and NAC and BaP co-treatment were detected by one-way analysis of variance (ANOVA). The abundance of mRNA and protein and the levels of apoptosis was expressed as mean ± SE (SD) and were analyzed by ANOVA followed by LSD (equal variances assumed) or Dunnett’s T3 (equal variances not assumed). A two-tailed *p* value of < 0.05 was considered statistically significant. Statistical analyses were conducted using SPSS 23.0 (IBM Co., NY, USA).

## Additional files

Additional file 1: **Table S1**. The PCR primer sequences in Sequenom EpiTYPER sequencing for human. **Table S2**. The PCR primer sequences in Sequenom EpiTYPER sequencing for mouse. **Table S3**. The sequences of primer for real-time PCR. **Table S4**. Methylation of *CASP8* gene using the Human Methylation 450 Bead Chip assay. **Table S5**. Demographic and obstetric characteristics of NTD cases and controls in Shanxi Province, China, 2011–2014. **Table S6**. Validation of differentially methylated CpG sites of *CASP8* gene in neural tissues of NTD cases and controls with Sequenom EpiTYPER. **Table S7**. Correlation analysis of differentially methylated CpG sites and PAH concentrations in maternal serum in NTD cases. **Table S8**. Correlation analysis of oxidative stress markers in fetal neural tissues and differentially methylated CpG sites in CASP8 in NTD cases. **Table S9**. Embryotoxicity of BaP and the effect of NAC in mouse embryo. **Table S10**. Differentially methylated CpG sites in *Casp8* in neural tissues of mouse embryo with and without BaP exposure/NAC rescue. (DOCX 65 kb)

Additional file 2: **Figure S1**. Head and crown–rump lengths of E10.5 mouse embryos exposed to BaP or BaP and NAC. **Figure S2**. NTDs in E10.5 mouse embryos exposed to BaP in vitro. **Figure S3**. The effects of BaP and NAC on apoptosis level of E10.5 embryos. **Figure S4**. Slice of E10.5 mouse embryo. (DOCX 1781 kb)

## Abbreviations

8-OHdG: 8-hydroxy-2′-deoxyguanosine
ad-*p*: Adjusted *p* value
ANOVA: One-way analysis of variance
BaP: Benz(a)pyrene
*CASP8/Casp8*: Caspase-8
Chr: Chromosome
CI: 95% confidence interval
DMSO: Dimethylsulphoxide
DNMT: DNA methyltransferase
E: Embryonic day
Fb: Forebrain
GPx: Glutathione peroxidase
H_PAHs: High-molecular-weight PAHs
Hb: Hindbrain
HM450K: Infinium HumanMethylation450 BeadChip
L_PAHs: Low-molecular-weight PAHs
Mb: Midbrain
MDA: Malondialdehyde
NAC: *N*-acetyl-L-cysteine
NTDs: Neural tube defects;
OR: Odds ratio
PAHs: Polycyclic aromatic hydrocarbons
PC: Protein carbonyl
ROS: Reactive oxygen species
SAM: *S*-adenosyl-L-methionine
SOD: Superoxide dismutase
TAC: Total antioxidant capacity
TET: Ten-eleven translocation
UTR: Untranslated region
