# Supplementary material for: Casp8 hypomethylation and neural tube defects in association with polycyclic aromatic hydrocarbon exposure
Source: Clin Epigenetics. 2019 May 7;11:72. doi: 10.1186/s13148-019-0673-6 (PMC6505285; doi:10.1186/s13148-019-0673-6)
Supplement: Supplementary file 1 — Table S1. The PCR primer sequences in Sequenom EpiTYPER sequencing for human. Table S2. The PCR primer sequences in Sequenom EpiTYPER sequencing for mouse. Table S3. The sequences of primer for real-time PCR. Table S4. Methylation of CASP8 gene using the Human Methylation 450 Bead Chip assay. Table S5. Demographic and obstetric characteristics of NTD cases and controls in Shanxi Province, China, 2011–2014. Table S6. Validation of differentially methylated CpG sites of CASP8 gene in neural tissues of NTD cases and controls with Sequenom EpiTYPER. Table S7. Correlation analysis of differentially methylated CpG sites and PAH concentrations in maternal serum in NTD cases. Table S8. Correlation analysis of oxidative stress markers in fetal neural tissues and differentially methylated CpG sites in CASP8 in NTD cases. Table S9. Embryotoxicity of BaP and the effect of NAC in mouse embryo. Table S10. Differentially methylated CpG sites in Casp8 in neural tissues of mouse embryo with and without BaP exposure/NAC rescue. (DOCX 65 kb) [file 13148_2019_673_MOESM1_ESM.docx]

# Additional file1

*Casp8* hypomethylation and neural tube defects in association with polycyclic aromatic hydrocarbon exposure

Yun Huang, Aiguo Ren, Linlin Wang, Lei Jin, Shanshan Lin, Zhiwen Li，Jasmine A. McDonald

**Table of Contents**

**Table s1.** The PCR primer sequences in Sequenom EpiTYPER sequencing for human.

**Table s2.** The PCR primer sequences in Sequenom EpiTYPER sequencing for mouse.

**Table s3.** The sequences of primer for real-time PCR.

**Table s4.** Methylation of *CASP8* gene using the Human Methylation 450 Bead Chip assay.

**Table s5.** Demographic and obstetric characteristics of NTD cases and controls in Shanxi Province, China, 2011–2014.

**Table s6.** Validation of differentially methylated CpG sites of *CASP8* gene in neural tissues of NTD cases and controls with Sequenom EpiTYPER.

**Table s7.** Correlation analysis of differentially methylated CpG sites and PAH concentrations in maternal serum in NTD cases.

**Table s8.** Correlation analysis of oxidative stress markers in fetal neural tissues and differentially methylated CpG sites in CASP8 in NTD cases.

**Table s9.** Embryotoxicity of BaP and the effect of NAC in mouse embryo.

**Table s10.** Differentially methylated CpG sites in *Casp8* in neural tissues of mouse embryo with and without BaP exposure/NAC rescue.

## **Table s1.** The PCR primer sequences in Sequenom EpiTYPER sequencing for human.

| Primer | Sequence |
| --- | --- |
| Amplicon_1F | aggaagagagGTGGTGGGTGTTTGTAGTTTTAGTT |
| Amplicon_1R | cagtaatacgactcactatagggagaaggctAATTCCCATCCTTAACCATATTCTC |
| Amplicon2_F | aggaagagagGATGGGAATTTAGTTTGAGTAGGGT |
| Amplicon2_R | cagtaatacgactcactatagggagaaggctAAAAAAAACTTCACCACAAAAAAAA |

## **Table s2.** The PCR primer sequences in Sequenom EpiTYPER sequencing for mouse.

| Primer | Sequence |
| --- | --- |
| Amplicon_1F | aggaagagagATTGAGAGAATTAGGGTATAGGGGAT |
| Amplicon_1R | cagtaatacgactcactatagggagaaggctTCACAAAAACTAAACCAACAATCAA |

## **Table s3.** The sequences of primer for real-time PCR.

| Primer | Sequence |
| --- | --- |
| *Casp8*_F | GGCCTCCATCTATGACCTGA |
| *Casp8*_R | TGTGGTTCTGTTGCTCGAAG |
| *Gapdh*_F | ATGACATCAAGAAGGTGGTG |
| *Gapdh*_R | CATACCAGGAAATGAGCTTG |

## **Table s4.** Methylation of *CASP8* gene using the Human Methylation 450 Bead Chip assay.

| Illumina ID | Chr | Mapinnfo | Case | Control | β-diff | ad-*p* | region |
| --- | --- | --- | --- | --- | --- | --- | --- |
| cg09464206 | 2 | 202122309 | 0.509 | 0.802 | -0.294 | 0.002 | TSS1500 |
| cg27410837 | 2 | 202122372 | 0.253 | 0.537 | -0.284 | 0.001 | TSS1500 |
| cg04048517 | 2 | 202097062 | 0.177 | 0.428 | -0.251 | 0.000 | TSS1500 |
| cg24410214 | 2 | 202097173 | 0.450 | 0.630 | -0.180 | 0.003 | TSS1500 |
| cg19448993 | 2 | 202097129 | 0.073 | 0.161 | -0.088 | 0.001 | TSS1500 |
| cg02878216 | 2 | 202097093 | 0.050 | 0.132 | -0.081 | 0.001 | TSS1500 |
| cg20608990 | 2 | 202097607 | 0.908 | 0.939 | -0.031 | 0.412 | TSS1500 |
| cg20435345 | 2 | 202121863 | 0.975 | 0.976 | -0.001 | 0.872 | TSS1500 |
| cg23882545 | 2 | 202122653 | 0.306 | 0.782 | -0.476 | 0.000 | TSS200 |
| cg14962032 | 2 | 202122669 | 0.120 | 0.535 | -0.414 | 0.000 | TSS200 |
| cg25748441 | 2 | 202122587 | 0.482 | 0.853 | -0.371 | 0.001 | TSS200 |
| cg25095814 | 2 | 202098016 | 0.774 | 0.910 | -0.136 | 0.023 | TSS200 |
| cg00978584 | 2 | 202122832 | 0.084 | 0.483 | -0.399 | 0.000 | 5'UTR |
| cg20418725 | 2 | 202122474 | 0.439 | 0.829 | -0.390 | 0.000 | 5'UTR |
| cg12604794 | 2 | 202122753 | 0.069 | 0.324 | -0.254 | 0.000 | 5'UTR |
| cg04286206 | 2 | 202123825 | 0.440 | 0.657 | -0.217 | 0.001 | 5'UTR |
| cg23061725 | 2 | 202126379 | 0.892 | 0.924 | -0.032 | 0.119 | 5'UTR |
| cg05130485 | 2 | 202098257 | 0.904 | 0.932 | -0.028 | 0.163 | 5'UTR |
| cg26842802 | 2 | 202125212 | 0.940 | 0.966 | -0.026 | 0.051 | 5'UTR |
| cg13109397 | 2 | 202101205 | 0.065 | 0.090 | -0.024 | 0.067 | 5'UTR |
| cg23817555 | 2 | 202124040 | 0.883 | 0.888 | -0.005 | 0.811 | 5'UTR |
| cg26799474 | 2 | 202098951 | 0.944 | 0.931 | 0.013 | 0.272 | 5'UTR |
| cg14930754 | 2 | 202121870 | 0.978 | 0.963 | 0.015 | 0.032 | 5'UTR |
| cg25073137 | 2 | 202124265 | 0.965 | 0.947 | 0.019 | 0.193 | 5'UTR |
| cg01466121 | 2 | 202136483 | 0.710 | 0.721 | -0.011 | 0.734 | Body |
| cg23281307 | 2 | 202142817 | 0.965 | 0.963 | 0.002 | 0.822 | Body |

Note: Target ID is identified according to Human Methylation 450 Bead Chip. The nucleotide position is based on NCBI build 37/hg19. Region is defined relative to the nearest open reading frame: within 1500 (TSS1500) or 200 bp (TSS200) of a transcription start site, in the 5’ UTR, the first exon of a transcript (exon) and in the body of gene (body). Differentially methylated CpG sites were identified by two criterions: the false discovery rate < 0.05, which was analyzed by independent t-tests with multiple comparison tests; the absolute *β*-value difference > 0.05.

Chr, chromosome; Mapinfo, nucleotide position; *β*-diff, difference of *β* value between NTD cases and controls; ad-*p*, adjusted *p*-value; UTR, untranslated region.

## **Table s5.** Demographic and obstetric characteristics of NTD cases and controls in Shanxi Province, China, 2011–2014.

| Characteristic | Cases | Controls*^a^* | *p* value*^b^* |
| --- | --- | --- | --- |
| Maternal age (y) |  |  | 0.563 |
| <25 | 34 (43.6) | 16 (55.2) |  |
| 25-29 | 21 (26.9) | 6 (20.7) |  |
| ≥30 | 23 (29.5) | 7 (24.1) |  |
| BMI (kg/m2) |  |  |  |
| <18.5 | 8 (10.5) | 2 (6.5) | 0.639 |
| 18.5-24.9 | 42 (55.3) | 20 (64.5) |  |
| ≥25 | 26 (34.2) | 9 (29.0) |  |
| Maternal education |  |  | 0.004 |
| Primary or lower | 9 (11.3) | 4 (12.5) |  |
| Junior high | 57 (71.3) | 13 (40.6) |  |
| High school or above | 14 (17.5) | 15 (46.9) |  |
| Occupation |  |  | <0.001 |
| Farmer | 67 (85.9) | 15 (46.9) |  |
| Non-farmer | 11 (14.1) | 17 (53.1) |  |
| Previous birth defects history |  |  | 0.577 |
| Yes | 4 (5.0) | 0 (0.0) |  |
| No | 76 (95.0) | 32 (100.0) |  |
| Gravidity |  |  | 0.146 |
| 1 | 32 (40.5) | 18 (56.3) |  |
| ≥2 | 47 (59.5) | 14 (43.8) |  |
| Parity |  |  | 0.028 |
| 1 | 33 (46.5) | 20 (71.4) |  |
| ≥2 | 38 (53.5) | 8 (28.6) |  |
| Unplanned pregnancy |  |  | 0.001 |
| Yes | 30 (38.0) | 23 (74.2) |  |
| No | 49 (62.0) | 8 (25.8) |  |
| Periconceptional folate supplementation |  |  | 0.001 |
| Yes | 47 (59.5) | 8 (25.0) |  |
| No | 32 (40.5) | 24 (75.0) |  |
| Fever or flu during early pregnancy |  |  | 0.112 |
| Yes | 28 (35.4) | 6 (18.8) |  |
| No | 51 (64.6) | 26 (81.3) |  |
| Active or passive smoking |  |  | 0.296 |
| Yes | 49 (61.3) | 16 (50.0) |  |
| No | 31 (38.8) | 16 (50.0) |  |
| Drinking |  |  | 1.000 |
| Yes | 3 (3.8) | 1 (3.1) |  |
| No | 77 (96.3) | 31 (96.9) |  |
| Primary fuel used for cooking |  |  | 1.000 |
| Coal | 13 (16.5) | 5 (15.6) |  |
| Natural gas/other | 66 (83.5) | 27 (84.4) |  |
| Primary fuel used for heating |  |  | 0.754 |
| Coal | 11 (13.9) | 3 (9.4) |  |
| Natural gas/other | 68 (86.1) | 29 (90.6) |  |
| Gestational age (weeks) |  |  | 0.169 |
| 13-27 | 56 (71.8) | 17 (56.7) |  |
| ≥28 | 22 (28.2) | 13 (43.3) |  |
| Fetus sex |  |  | 0.287 |
| Male | 35 (47.9) | 18 (60.0) |  |
| Female | 38 (52.1) | 12 (40.0) |  |

*^a^* The total number may not be equal to the number of cases or controls due to missing or unknown data.

*^b^* The χ^2^ test or Fisher's exact test were used to compare demographic information between the case and control groups.

NTD, neural tube defect.

## **Table s6.** Validation of differentially methylated CpG sites of *CASP8* gene in neural tissues of NTD cases and controls with Sequenom EpiTYPER.

| CpG site | Cases | | |  |  | Controls | |  | Difference | | | | *p* value |
| --- | --- | --- | --- | --- | --- | --- | --- | --- | --- | --- | --- | --- | --- |
|  | N | Mean | SD |  | N | Mean | SD |  | Mean | Std. Error | 95% Confidence Interval | |  |
| *CASP8*_CpG_1 | 77 | 0.041 | 0.051 |  | 32 | 0.183 | 0.140 |  | 0.142 | 0.025 | 0.091 | 0.194 | 0.000 |
| *CASP8*_CpG_2 | 80 | 0.194 | 0.112 |  | 32 | 0.457 | 0.271 |  | 0.263 | 0.050 | 0.162 | 0.364 | 0.000 |
| *CASP8*_CpG_3 | 80 | 0.358 | 0.133 |  | 32 | 0.605 | 0.232 |  | 0.247 | 0.044 | 0.159 | 0.335 | 0.000 |
| *CASP8*_CpG_4 | 80 | 0.301 | 0.154 |  | 32 | 0.547 | 0.268 |  | 0.246 | 0.050 | 0.144 | 0.348 | 0.000 |
| *CASP8*_CpG_5 | 80 | 0.421 | 0.158 |  | 32 | 0.607 | 0.221 |  | 0.186 | 0.043 | 0.099 | 0.272 | 0.000 |
| *CASP8*_CpG_6 | 80 | 0.634 | 0.148 |  | 32 | 0.722 | 0.133 |  | 0.088 | 0.030 | 0.028 | 0.147 | 0.004 |
| *CASP8*_CpG_7 | 78 | 0.618 | 0.155 |  | 32 | 0.710 | 0.112 |  | 0.092 | 0.030 | 0.032 | 0.152 | 0.003 |

Note: analyzed by independent t-tests. SD, standard deviation; Std Error, standard error.

## **Table s7.** Correlation analysis of differentially methylated CpG sites and PAH concentrations in maternal serum in NTD cases.

| CpG site | N | Total PAHs, | |  | L_PAHs, | |  | H_PAHs, | |
| --- | --- | --- | --- | --- | --- | --- | --- | --- | --- |
|  |  | ng/g lipid | |  | ng/g lipid | |  | ng/g lipid | |
|  |  | ρ | *p* |  | ρ | *p* |  | ρ | *p* |
| CASP8_CpG_1 | 50 | -0.095 | 0.511 |  | -0.087 | 0.546 |  | -0.113 | 0.435 |
| CASP8_CpG_2 | 52 | -0.203 | 0.148 |  | -0.199 | 0.157 |  | -0.190 | 0.177 |
| CASP8_CpG_3 | 52 | -0.144 | 0.307 |  | -0.133 | 0.346 |  | -0.169 | 0.232 |
| CASP8_CpG_4 | 52 | -0.222 | 0.114 |  | -0.213 | 0.130 |  | -0.226 | 0.107 |
| CASP8_CpG_5 | 52 | -0.224 | 0.111 |  | -0.204 | 0.147 |  | -0.271 | 0.052 |
| CASP8_CpG_6 | 52 | -0.052 | 0.713 |  | -0.017 | 0.906 |  | -0.194 | 0.169 |
| CASP8_CpG_7 | 50 | -0.239 | 0.094 |  | -0.220 | 0.125 |  | -.284^*^ | 0.045 |
| CASP8_average | 52 | -0.230 | 0.100 |  | -0.210 | 0.135 |  | -.282^*^ | 0.043 |

Note: PAHs, polycyclic aromatic hydrocarbons; total PAHs, sum of all PAHs; L_PAHs, sum of low-molecular-weight PAHs, including acenaphthylene, acenaphthene, fluorene, phenanthrene, anthracene, fluoranthene, and retene; H_PAHs, sum of high-molecular-weight PAHs, including pyrene, benz[a]anthracene, chrysene, benzo[b]fluoranthene, benzo[k]fluoranthene, and benzo[a]pyrene; ρ, Pearson’s correlation coefficient.

## **Table s8.** Correlation analysis of oxidative stress markers in fetal neural tissues and differentially methylated CpG sites in CASP8 in NTD cases.

| CpG site | N | SOD, unit/mg protein | |  | GPx, unit/mg protein | |  | TAC, unit/mg protein | |  | MDA, nmol/mg protein | |  | PC, nmol/mg protein | |
| --- | --- | --- | --- | --- | --- | --- | --- | --- | --- | --- | --- | --- | --- | --- | --- |
|  |  |  |  |  |  |  |  |  |  |  |  |  |  |  |  |
|  |  | *ρ* | *p* |  | *ρ* | *p* |  | *ρ* | *p* |  | *ρ* | *p* |  | *ρ* | *p* |
| *CASP8*_CpG_1 | 20 | 0.069 | 0.774 |  | -0.356 | 0.124 |  | -0.073 | 0.759 |  | -0.208 | 0.379 |  | -0.403 | 0.078 |
| *CASP8*_CpG_2 | 20 | 0.195 | 0.409 |  | -0.220 | 0.352 |  | 0.032 | 0.893 |  | -0.225 | 0.340 |  | -0.228 | 0.334 |
| *CASP8*_CpG_3 | 20 | 0.143 | 0.548 |  | -0.329 | 0.157 |  | 0.140 | 0.557 |  | -0.108 | 0.649 |  | -0.292 | 0.211 |
| *CASP8*_CpG_4 | 20 | -0.046 | 0.848 |  | -0.337 | 0.146 |  | 0.037 | 0.876 |  | -0.121 | 0.612 |  | -0.259 | 0.270 |
| *CASP8*_CpG_5 | 20 | 0.007 | 0.976 |  | -0.376 | 0.102 |  | 0.065 | 0.786 |  | -0.088 | 0.713 |  | -0.123 | 0.604 |
| *CASP8*_CpG_6 | 20 | 0.113 | 0.636 |  | -.547 | 0.013 |  | 0.081 | 0.734 |  | -0.020 | 0.934 |  | 0.001 | 0.998 |
| *CASP8*_CpG_7 | 19 | -0.446 | 0.056 |  | -.727 | 0.000 |  | 0.311 | 0.194 |  | 0.364 | 0.125 |  | -0.290 | 0.228 |
| *CASP8*_average | 20 | -0.015 | 0.950 |  | -.462 | 0.041 |  | 0.127 | 0.593 |  | -0.113 | 0.634 |  | -0.235 | 0.319 |

Note: SOD, superoxide dismutase; GPx, glutathione peroxidase; TAC, total antioxidant capacity; MDA, malondialdehyde; PC, protein carbonyl; ρ, Pearson’s correlation coefficient.

## **Table s9.** Embryotoxicity of BaP and the effect of NAC in mouse embryo.

| Group | N | Viable embryos | Viability rate (%) | NTDs embryos | NTDs rate (%) |
| --- | --- | --- | --- | --- | --- |
| DMSO | 38 | 38 | 100 | 0 | 0 |
| BaP | 37 | 37 | 100 | 5 | 13.5*^*#^* |
| BaP+NAC | 42 | 42 | 100 | 0 | 0 |

*^*^* Significant difference from vehicle-treated embryos (*p* < 0.05).

*^#^* Significant difference from NAC co-treated (5uM) embryos (*p* < 0.05).

## **Table s10.** Differentially methylated CpG sites in *Casp8* in neural tissues of mouse embryo with and without BaP exposure/NAC rescue.

| **CpG site** | **DMSO controls (1)** | | |  | **BaP (2)** | | |  | **BaP+NAC (3)** | | | **Difference between (1) and (2)** | **Difference between (2) and (3)** | **Difference between (1) and (3)** |
| --- | --- | --- | --- | --- | --- | --- | --- | --- | --- | --- | --- | --- | --- | --- |
|  | **N** | **Mean** | **SD** |  | **N** | **Mean** | **SD** |  | **N** | **Mean** | **SD** | ***p* value** | ***p* value** | ***p* value** |
| *Casp8*_CpG_1 | 21 | 0.192 | 0.193 |  | 22 | 0.076 | 0.08 |  | 22 | 0.148 | 0.113 | 0.007 | 0.085 | 0.291 |
| *Casp8*_CpG_2 | 20 | 0.075 | 0.105 |  | 20 | 0.016 | 0.025 |  | 21 | 0.077 | 0.088 | 0.025 | 0.019 | 0.932 |
| *Casp8*_CpG_3 | 21 | 0.166 | 0.098 |  | 21 | 0.105 | 0.073 |  | 22 | 0.112 | 0.055 | 0.013 | 0.765 | 0.025 |
| *Casp8*_CpG_4 | 21 | 0.101 | 0.056 |  | 21 | 0.059 | 0.03 |  | 22 | 0.11 | 0.054 | 0.006 | 0.001 | 0.543 |
| *Casp8*_CpG_5 | 21 | 0.429 | 0.149 |  | 21 | 0.332 | 0.124 |  | 22 | 0.423 | 0.105 | 0.016 | 0.022 | 0.871 |

Note: differentially methylated CpG sites of *Casp8* were detected by one-way analysis of variance.
