# Supplementary material for: Casp8 hypomethylation and neural tube defects in association with polycyclic aromatic hydrocarbon exposure
Source: Clin Epigenetics. 2019 May 7;11:72. doi: 10.1186/s13148-019-0673-6 (PMC6505285; doi:10.1186/s13148-019-0673-6)
Supplement: Supplementary file 2 — Figure S1. Head and crown–rump lengths of E10.5 mouse embryos exposed to BaP or BaP and NAC. Figure S2. NTDs in E10.5 mouse embryos exposed to BaP in vitro. Figure S3. The effects of BaP and NAC on apoptosis level of E10.5 embryos. Figure S4. Slice of E10.5 mouse embryo. (DOCX 1781 kb) [file 13148_2019_673_MOESM2_ESM.docx]

# Additional file2

*Casp8* hypomethylation and neural tube defects in association with polycyclic aromatic hydrocarbon exposure

Yun Huang, Aiguo Ren, Linlin Wang, Lei Jin, Shanshan Lin, Zhiwen Li, Jasmine A. McDonald

**Table of Contents**

**Figure s1.** Head and crown-rump lengths of E10.5 mouse embryos exposed to BaP or BaP and NAC.

**Figure s2.** NTDs in E10.5 mouse embryos exposed to BaP in vitro.

**Figure s3.** The effects of BaP and NAC on apoptosis level of E10.5 embryos.

**Figure s4.** Slice of E10.5 mouse embryo.


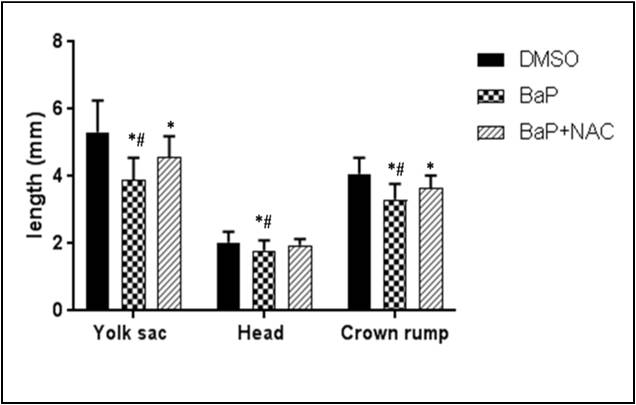


## **Figure s1.** Head and crown-rump lengths of E10.5 mouse embryos exposed to BaP or BaP and NAC. Data were shown as mean ± SD, Error bars represent SD. DMSO, dimethylsulphoxide; BaP, benz(a)pyrene; NAC, N-acetyl-L-cysteine; SD, standard deviation.


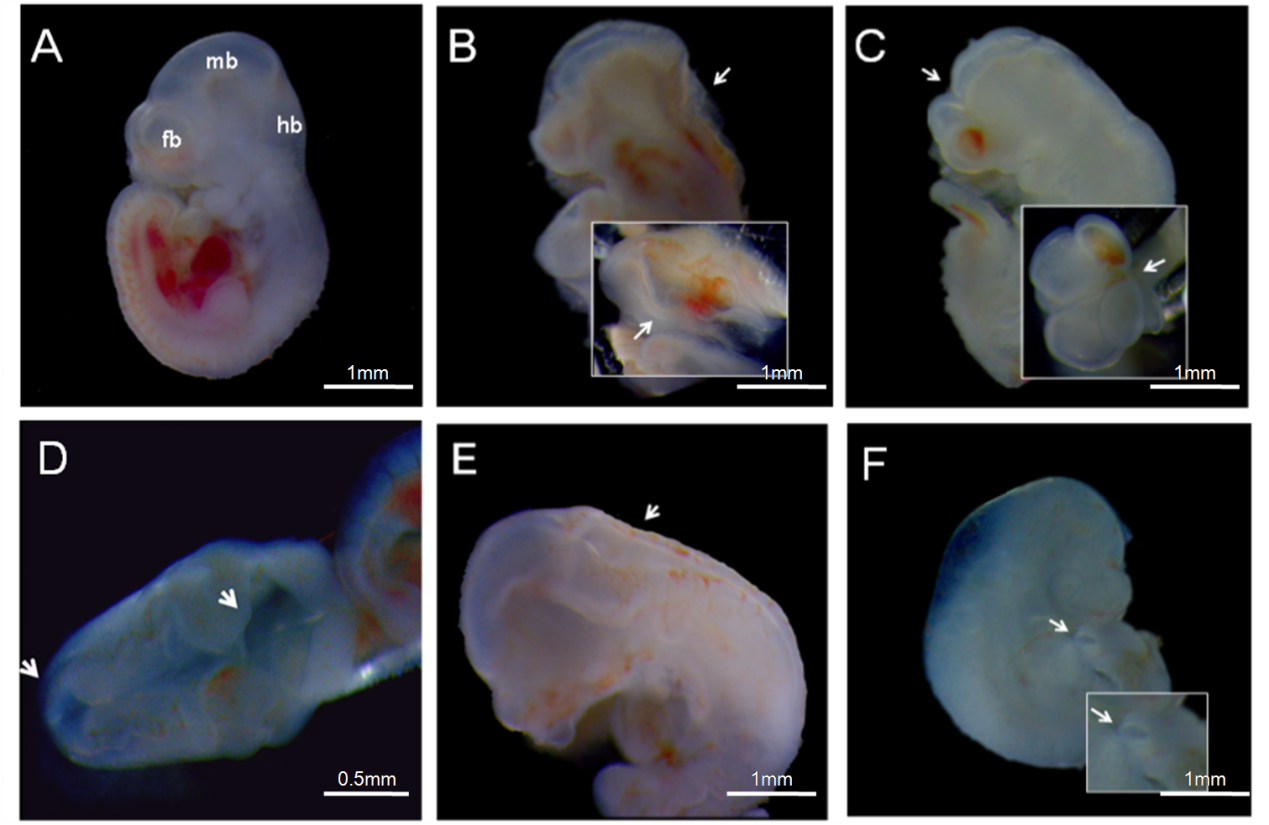


## **Figure s2.** NTDs in E10.5 mouse embryos exposed to BaP in vitro.

A. Control E10.5 embryo; fb, forebrain; mb, midbrain; hb, hindbrain.

B. Embryo with unfused neural folds resulting in craniorachischisis (arrow).

C. Embryo with closure failure in cranial region resulting in anencephaly (arrow).

D. Embryo with closure failure near the forebrain and midbrain resulting in exencephaly (arrow).

E. Embryo with open lesions of spinal regions resulting in open spina bifida (arrow);

F. Embryo with closure failure in posterior neuropore resulting in lumbosacral spina bifida (arrow).


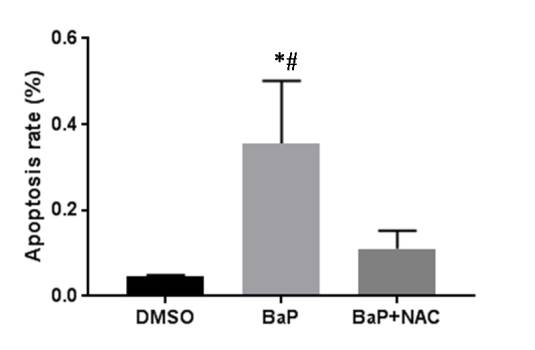


## **Figure s3.** The effects of BaP and NAC on apoptosis level of E10.5 embryos. Quantitative results of the TUNEL assay for E10.5 mouse embryos exposed to BaP and co-exposed to NAC. Apoptotic cells were counted and expressed as a percentage of total cell number. Error bars represent standard error of the mean. DMSO, dimethylsulphoxide; BaP, benz(a)pyrene; NAC, N-acetyl-L-cysteine.

##
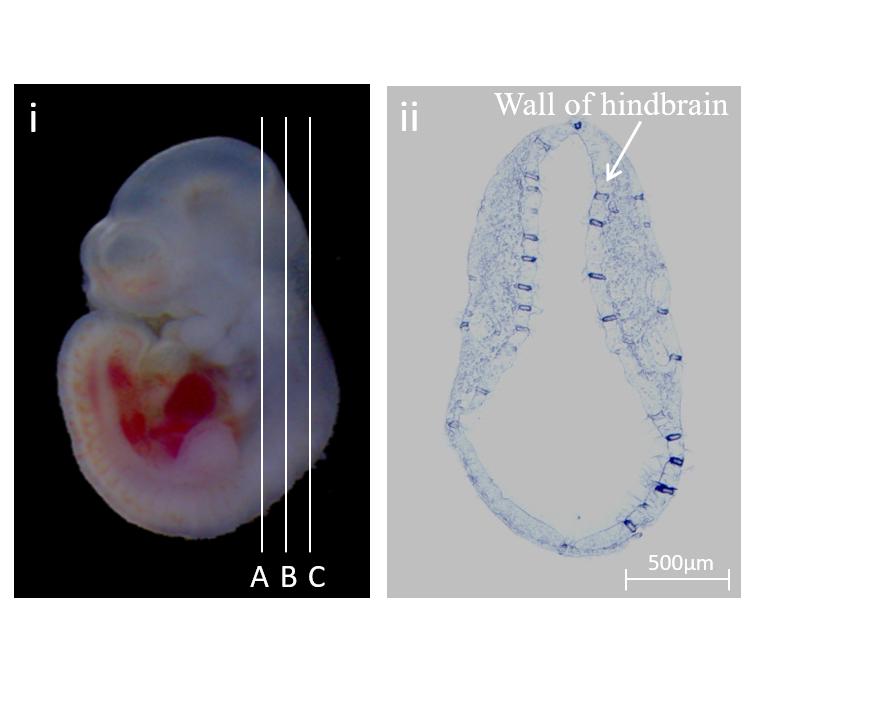


## **Figure s4.** Slice of E10.5 mouse embryo. i Slice direction; ii Representative image of slice chosen for TUNEL assay.
